# Supplementary figures and images for: Selective suppression of oligodendrocyte-derived amyloid beta rescues neuronal dysfunction in Alzheimer’s disease
Source: PLoS Biol. 2024 Jul 23;22(7):e3002727. doi: 10.1371/journal.pbio.3002727 (PMC11265669; doi:10.1371/journal.pbio.3002727)

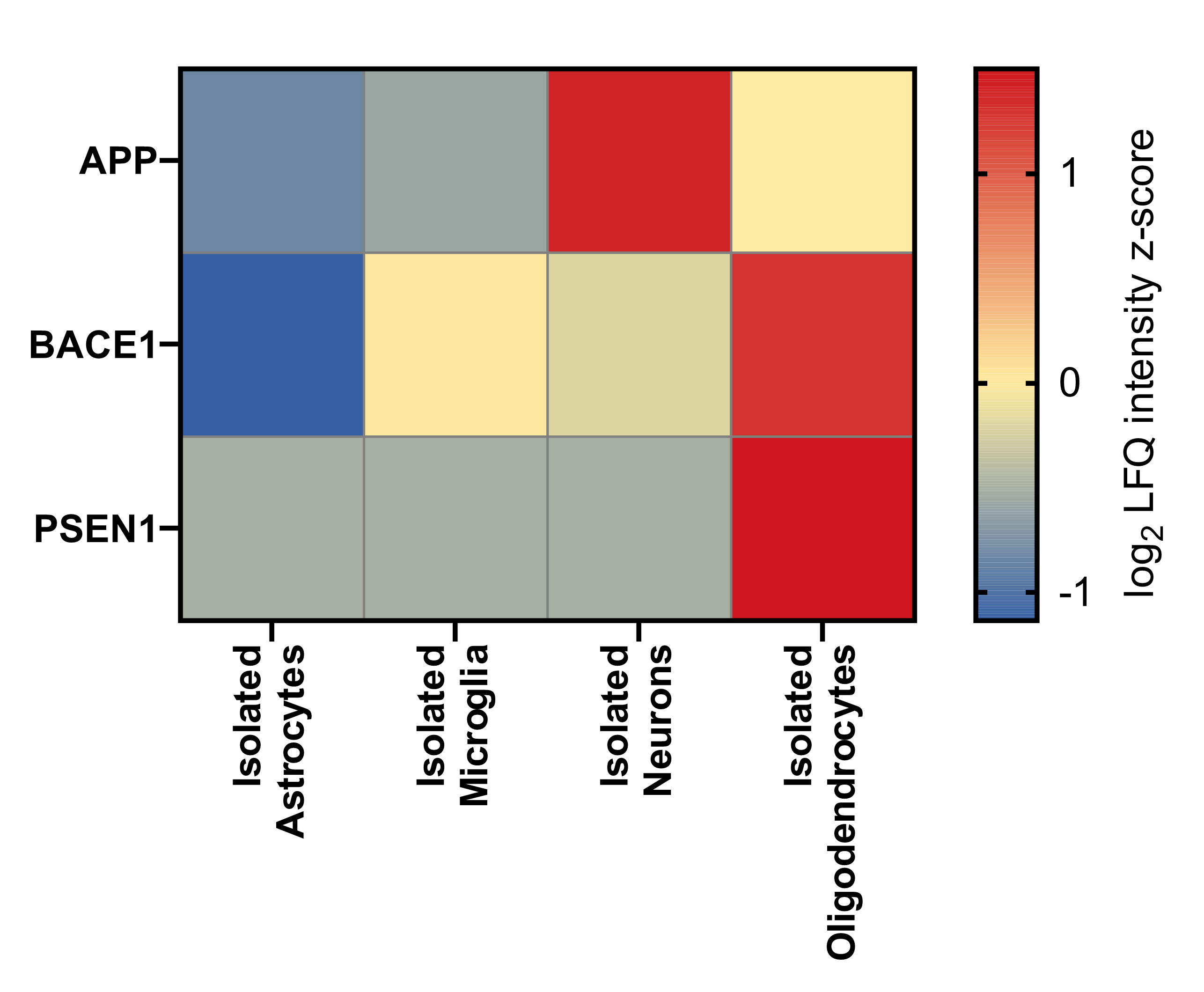

Supplement: S1 Fig — Heatmap showing the log2 (LFQ intensity) z-score of proteins of interest from isolated mouse astrocytes, microglia, neurons, and oligodendrocytes shows high amounts of APP, BACE1, and PSEN1 in isolated oligodendrocytes. Proteomics data from Sharma and colleagues [14] was generated from cells isolated by Magnetic-Activated Cell Sorting (MACS) from C57/BL6 mice. (TIF) [file pbio.3002727.s001.tif]

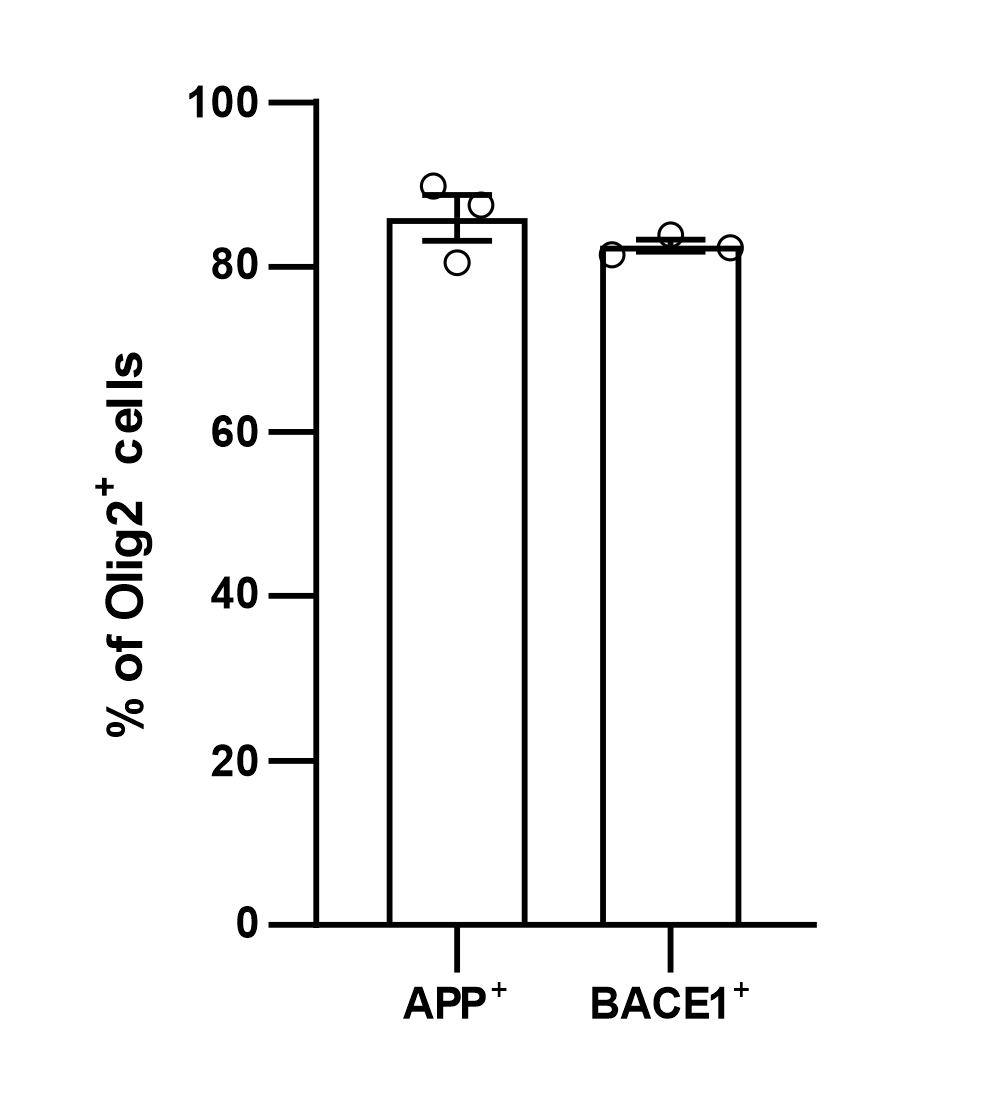

Supplement: S2 Fig — Quantification of immunofluorescent images from 4-month-old wild-type mice (see Fig 1E and 1F) showing the percentage of Olig2+ cells which are APP+ or BACE1+. Each data point represents an individual mouse (n = 3) with bars showing mean ± SEM. Source data are available in S1 Data. (TIF) [file pbio.3002727.s002.tif]

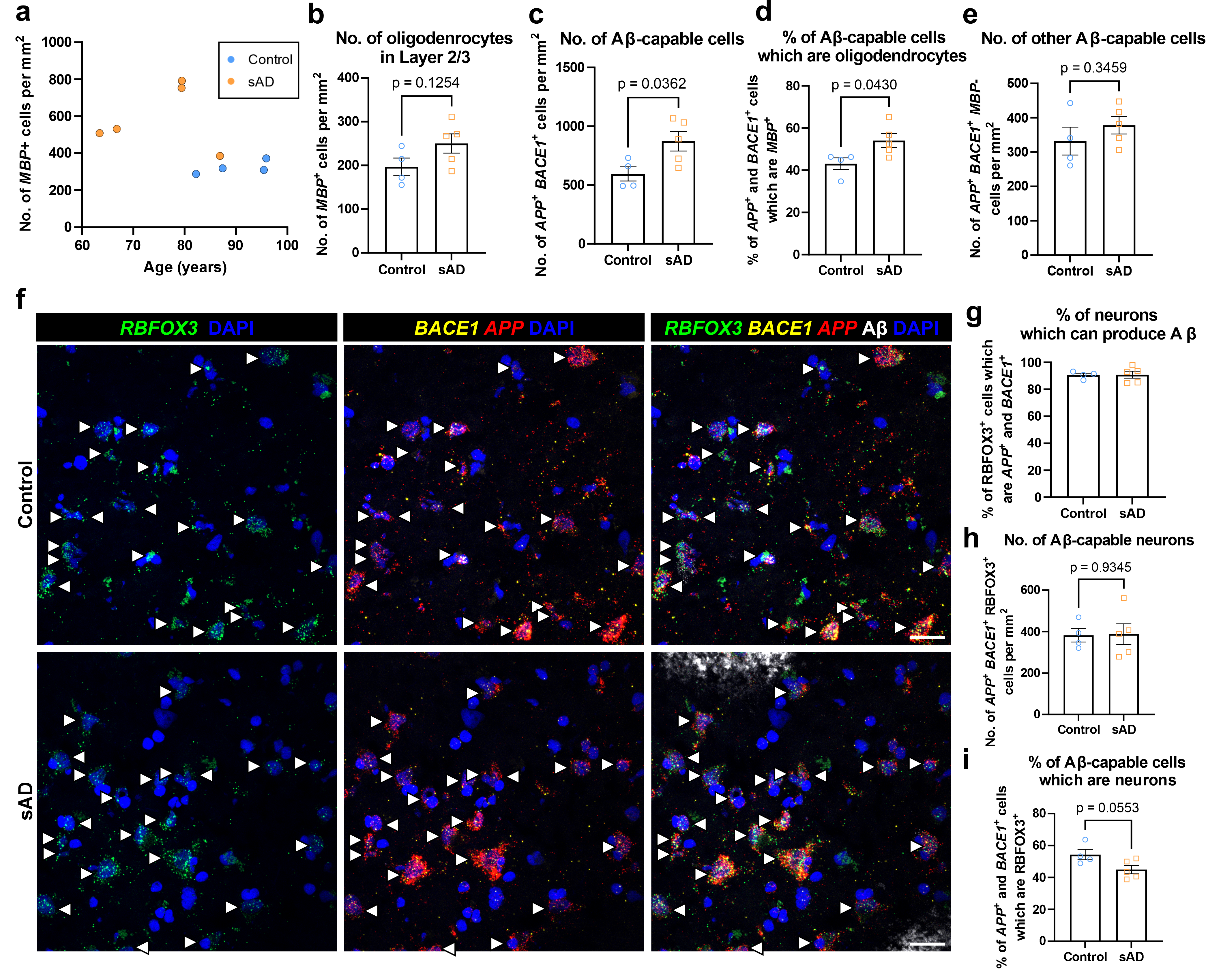

Supplement: S3 Fig — (a) Plot showing the number of MBP+ cells found in Layers 5/6 (Fig 1A and 1C) of the prefrontal cortex against the age of the donor. A linear regression analysis indicated no significant relationship (F(1,7) = 1.958, p = 0.2045), with a low coefficient of determination (r2 = 0.2185), suggesting that age is not a significant contributor to the number of MBP+ cells in Layers 5/6 of the prefrontal cortex. (b) Quantification showing no significant change in the number of oligodendrocytes in Layer 2/3 of the prefrontal cortex of AD brains. (c) Quantification showing a significant increase in the number of all Aβ producing cells in sporadic AD (sAD) brains compared to controls. (d) Quantification showing a significant increase in the proportion of Aβ producing cells which are oligodendrocytes in sAD brains. (e) Quantification of the number of Aβ producing cells which are not oligodendrocytes showing no significant difference between control and AD brains. (f) Fluorescent images from Layers 5/6 of control (top) and sporadic AD (sAD; bottom) postmortem human prefrontal cortex labelled for RBFOX3 (neuron-specific gene; green), BACE1 (yellow), APP (red), Aβ (identified by 6E10-antibody; white), and DAPI (nuclei; blue). Aβ-capable neurons (RBFOX3+ BACE1+ APP+ nuclei) are marked with white arrowheads. Note the high variability in expression levels of APP and BACE1 between neurons, with high expression in some cells but minimal expression in others. Scale bar = 25 μm. (g) Quantification of the proportion of neurons which are capable of producing Aβ. (h) Quantification of the number of neurons which are capable of producing Aβ. (i) Quantification showing the proportion of Aβ-capable cells which are neurons. Each data point represents a single brain (n = 4 control brains, n = 5 sAD brains) with bars representing mean ± SEM. Unpaired t test; t(7) = 1.740, 2.585, 2.467, 1.011, 0.08304, 0.08520, 2.297 in (b), (c), (d), (e), (g), (h), and (i), respectively. Source data are available i [file pbio.3002727.s003.tif]

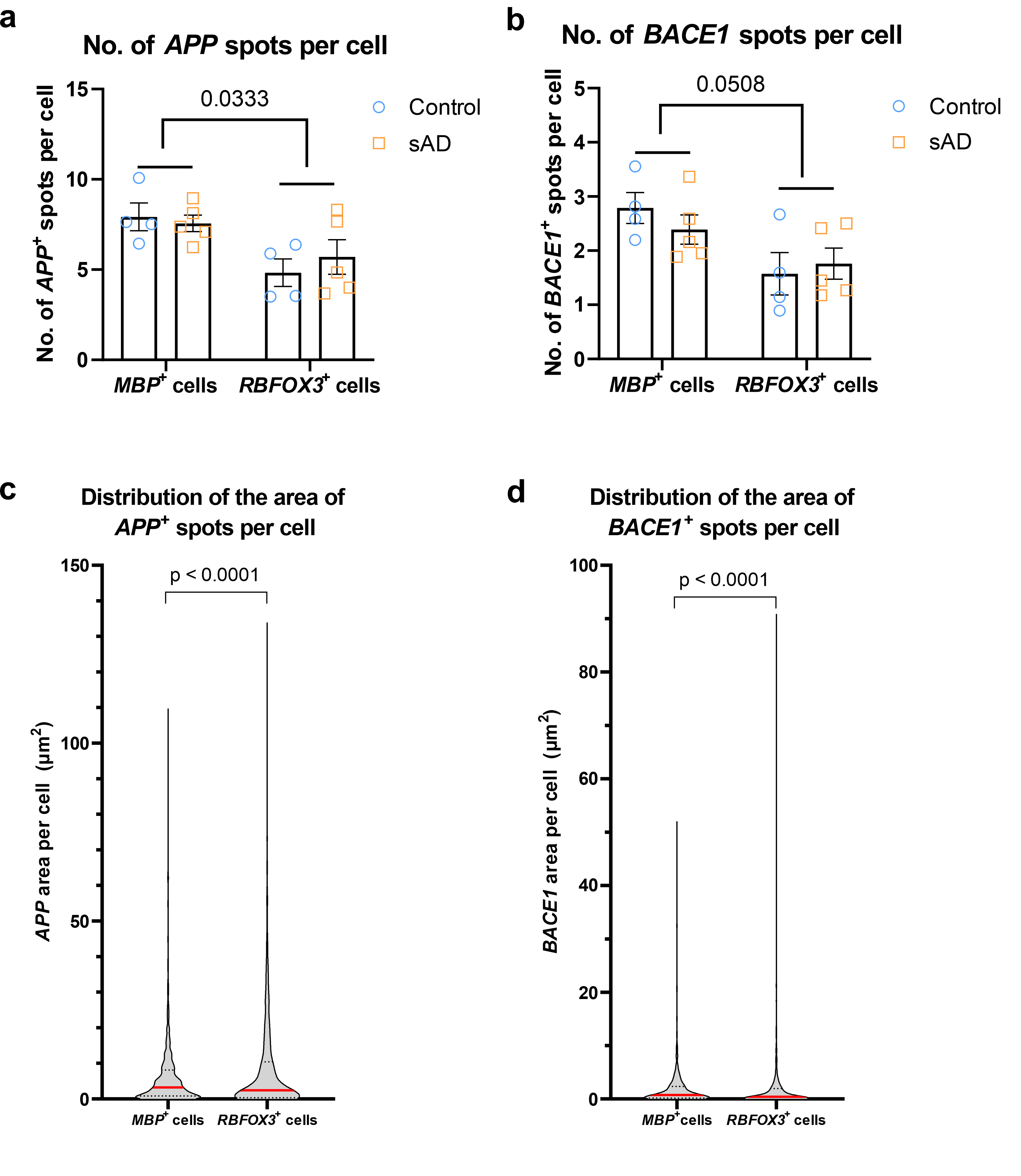

Supplement: S4 Fig — (a, b) Quantification showing more APP+ (a) and BACE1+ (b) spots per cell in oligodendrocytes (MBP+ cells) compared to neurons (RBFOX3+ cells) in both control and sAD human postmortem brains. Each data point represents a single brain (n = 4 control brains, n = 5 sAD brains) with bars representing mean ± SEM. Two-way repeated measures ANOVA. Cell type effect: (a) F(1,7) = 6.979; (b) F(1,7) = 5.540. (c, d) Violin plots demonstrating the greater variability in expression levels of APP (c) and BACE1 (d) in neurons (RBFOX3+ cells) compared to oligodendrocytes (MBP+ cells) in human postmortem brains (n = 843 MBP+ cells, n = 1,100 RBFOX3+ cells from 4 control and 5 sAD brains). Red lines indicate mean, while dotted black lines indicate quartiles. Fligner–Killeen test for equality of variances: FK(1,1944) = 388.69, 533.44 in (c and d), respectively. Source data are available in S1 Data. (TIF) [file pbio.3002727.s004.tif]

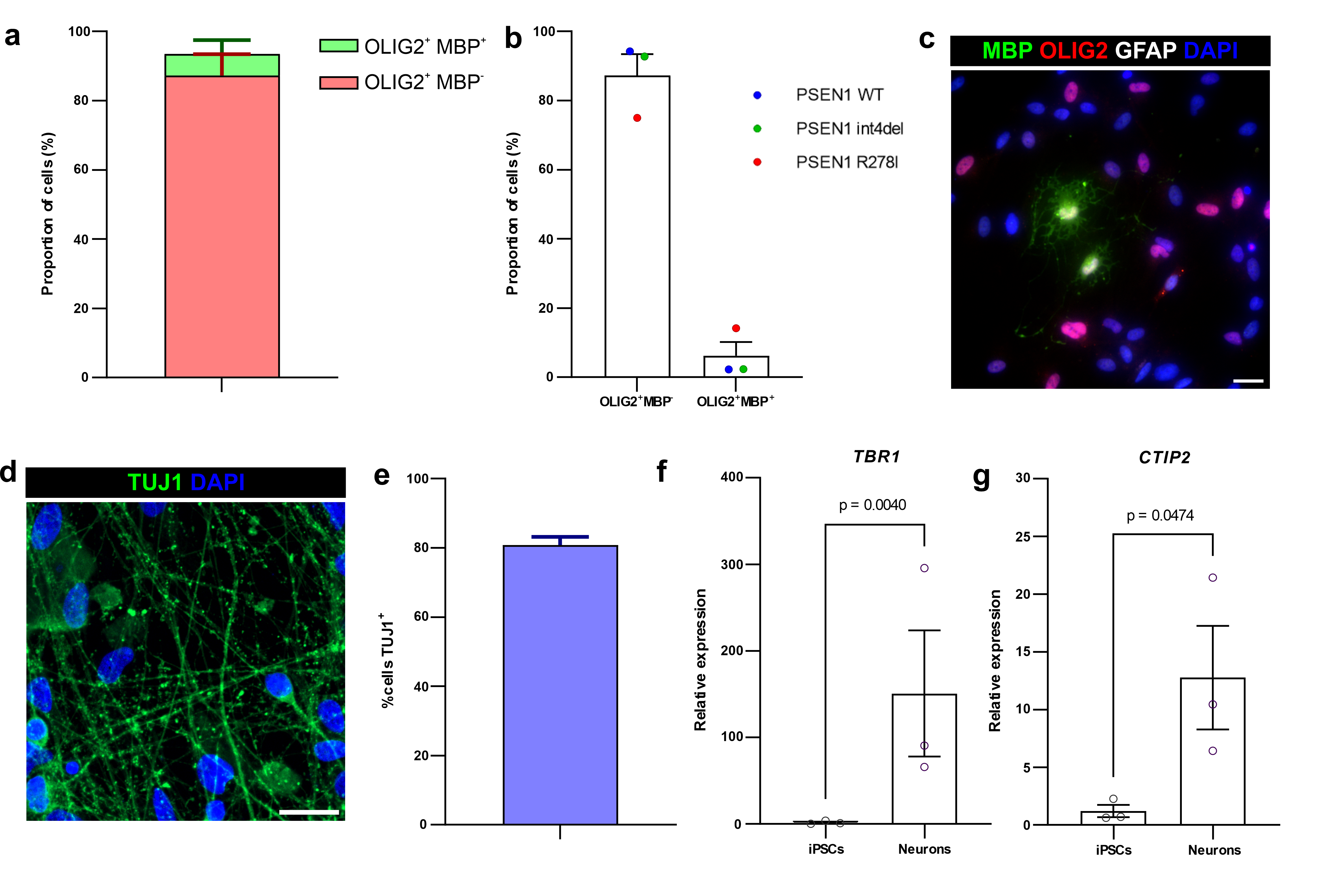

Supplement: S5 Fig — (a) Quantification of the proportion of cells in oligodendrocyte cultures which are oligodendrocyte precursor cells (OPCs; OLIG2+ MBP-) and mature, myelin-expressing oligodendrocytes (OLIG2+ MBP+) shows that 93.5% ± 2.2% of all cells in these cultures are either oligodendrocytes or OPCs. Bars show mean + SEM. n = 3 cell lines (1 induction per line). (b) Separated data from (a) showing the quantification of the proportion of cells in oligodendrocyte cultures which OPCs (OLIG2+ MBP-) and mature, myelin-expressing oligodendrocytes (OLIG2+ MBP+). Each data point represents an independent differentiation (n = 1 per line) with data from each line shown in a different colour, and bars showing mean + SEM. (c) Representative fluorescent image of human iPSC-derived oligodendrocyte culture immunolabelled for MBP (green), OLIG2 (red), GFAP (white), and DAPI (blue). No GFAP+ astrocytes were found in 3 out of 3 cultures examined. Scale bar = 25 μm. (d) Fluorescent image of human iPSC-derived neuronal culture immunolabelled for the neuronal marker TUJ1 (green) and DAPI (nuclei; blue). Scale bar = 25 μm. (e) Quantification of the proportion of cells in neuronal cultures which are neurons shows a high proportion of TUJ1+ cells, consistent with previous studies [75]. Bar shows mean + SEM. n = 3 cell lines (1 induction per line). (f, g) qPCR data showing high expression of deep cortical layer markers TBR1 (f) and CTIP2 (g) in neuronal cultures compared to undifferentiated iPSCs. Graphs show relative expression of the gene of interest, normalised to the average for iPSC cultures using the ΔΔCt method (with RPL18A as the housekeeping gene). Each data point represents a different cell line (n = 3; 1 induction per line) with bars showing mean ± SEM. Ratio paired t test: t(2) = 15.73, 4.430 in (f and g), respectively. Source data are available in S1 Data. (TIF) [file pbio.3002727.s005.tif]

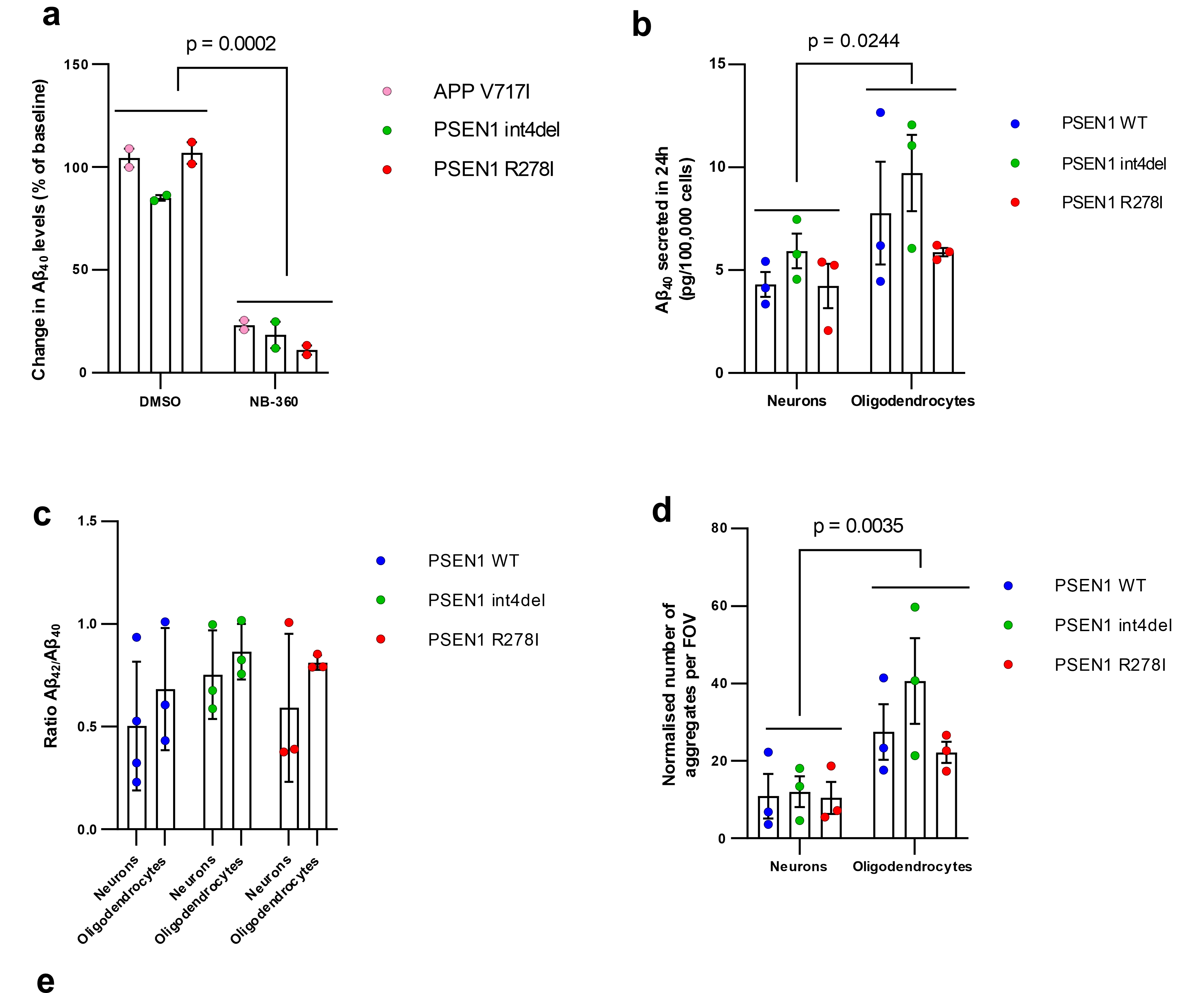

Supplement: S6 Fig — (a) Un-pooled data from Fig 3B: quantification by ELISA showing a significant reduction in the amount of Aβ40 produced (as a % of the amount produced prior to treatment) by human oligodendrocytes when treated with BACE1 inhibitor (NB-360) compared to vehicle control (DMSO). Each data point represents an independent differentiation (n = 3 per line) with data from each line shown in a different colour and bars showing mean ± SEM. Two-way repeated measures ANOVA: Treatment effect: F(1,3) = 468.0, p = 0.0002; Cell line effect: F(2,3) = 5.987, p = 0.0897. (b) Un-pooled data from Fig 3C: ELISA data showing more Aβ40 produced by oligodendrocytes than neurons derived from the same human-iPSC lines. Each data point represents an independent differentiation (n = 3 per line) with data from each line shown in a different colour and bars showing mean ± SEM. Two-way ANOVA: Cell type effect: F(1,12) = 6.622, p = 0.0244; Cell line effect: F(2,12) = 1.984, p = 0.1802. (c) Un-pooled data from Fig 3D: quantification by ELISA showing higher Aβ42/Aβ40 ratio produced by oligodendrocytes compared to neurons derived from the same fAD human-iPSC lines. Each data point represents an independent differentiation [n = 4(PSEN1 WT neurons) or 3 per line] with data from each line shown in a different colour and bars showing mean ± SEM. Two-way ANOVA: Cell type effect: F(1,13) = 2.058, p = 0.1750; Cell line effect: F(2,13) = 1.131, p = 0.3525. (d) Un-pooled data from Fig 3F: quantification showing oligodendrocytes produce a higher proportion of Aβ as aggregates compared to neurons derived from the same human-iPSC lines. Each data point represents an independent differentiation (n = 3 per line) with data from each line shown in a different colour and bars showing mean ± SEM. Two-way ANOVA: Cell type effect: F(1,12) = 13.10, p = 0.0035; Cell line effect: F(2,12) = 1.289, p = 0.3112. Source data are available in S1 Data. (TIF) [file pbio.3002727.s006.tif]

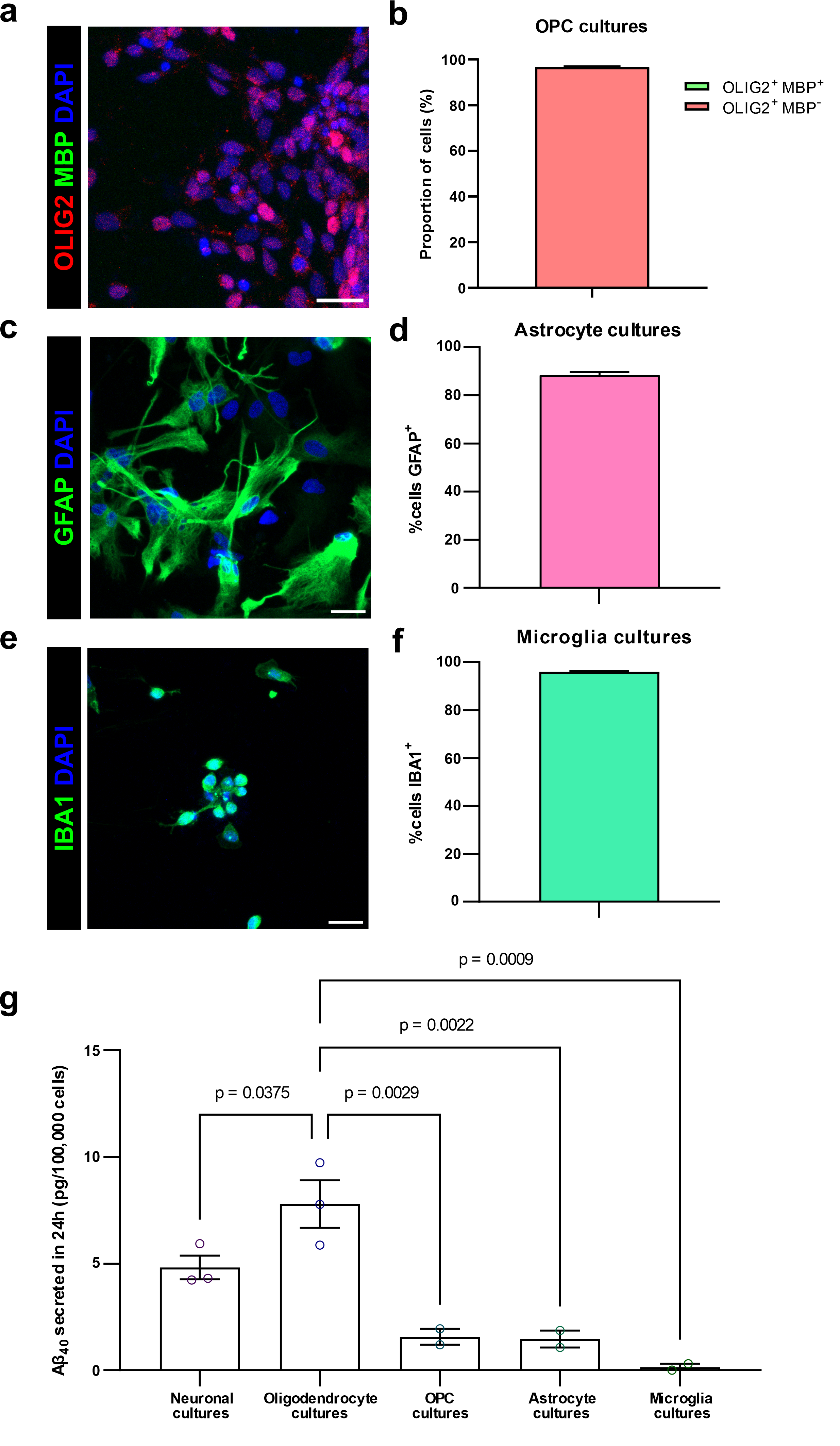

Supplement: S7 Fig — (a–f) Representative fluorescent images (a, c, e) and quantifications showing characterisation of OPC (a, b), astrocyte (c, d), and microglia (e, f) cultures. In (a), cells are immunolabelled for MBP (myelin basic protein; green), OLIG2 (marker of all oligodendroglia; red), and DAPI (nuclei; blue), with quantification in (b) showing 96.8% ± 0.2% of cells are OLIG2+ MBP- OPCs while there are no mature MBP+ oligodendrocytes. In (c), cells are immunolabelled for GFAP (green) and DAPI (blue), with quantification in (d) showing 88.3% ±1.0% of cells are GFAP+ astrocytes. In (e), cells are immunolabelled for IBA1 (green) and DAPI (blue), with quantification in (f) showing 95.9% ± 0.2% of cells are IBA1+ microglia. In (a, c, e), scale bar = 25 μm. In (b, d, f) bars show mean + SEM, n = 2 cell lines (1 induction per line). (g) ELISA data showing very low amounts of Aβ40 produced by human iPSC-derived OPCs, astrocytes, and microglia compared to neurons and oligodendrocytes. Each data point represents the average of 2 (OPCs, astrocytes, microglia) or 3 (neurons, oligodendrocytes) independent inductions/harvests from a different cell line (OPCs, astrocytes, microglia: n = 2; neurons, oligodendrocytes: n = 3), with bars representing mean ± SEM. Mixed effects analysis (F(4,5) = 27.77, p = 0.013) with Dunnet’s post hoc tests. Source data are available in S1 Data. (TIF) [file pbio.3002727.s007.tif]

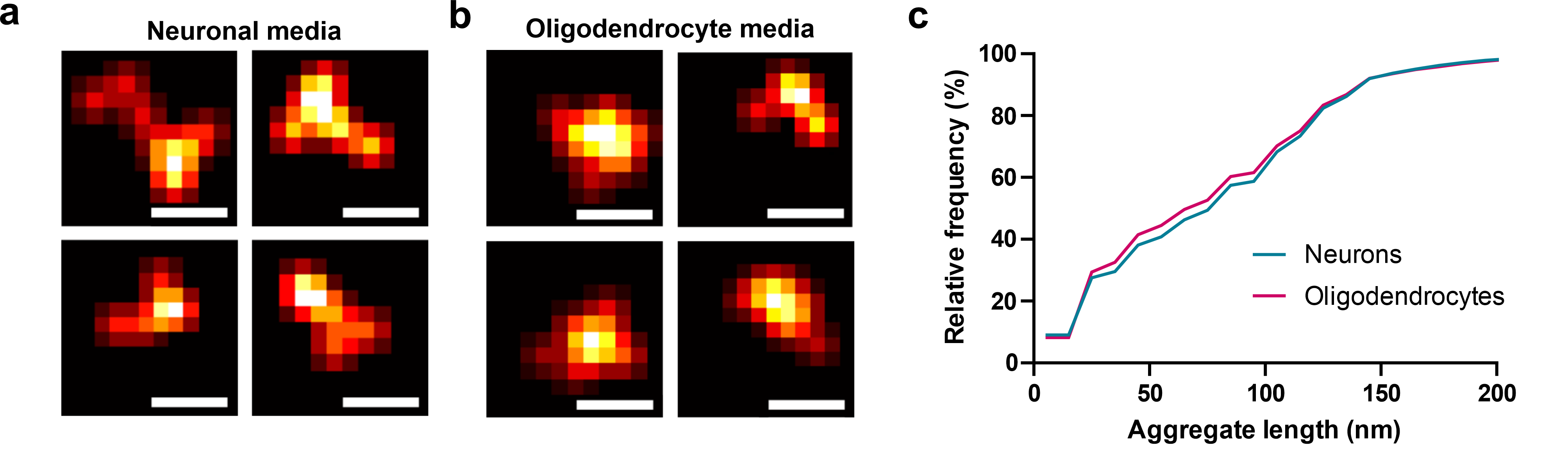

Supplement: S8 Fig — (a, b) Examples of super-resolved aggregates detected in neuronal media (a) and oligodendrocyte media (b). Scale bar = 50 nm. (c) Cumulative frequency histogram showing the size distribution of aggregates produced by neurons and oligodendrocytes (bin size = 10 nm). Source data are available in S1 Data. (TIF) [file pbio.3002727.s008.tif]

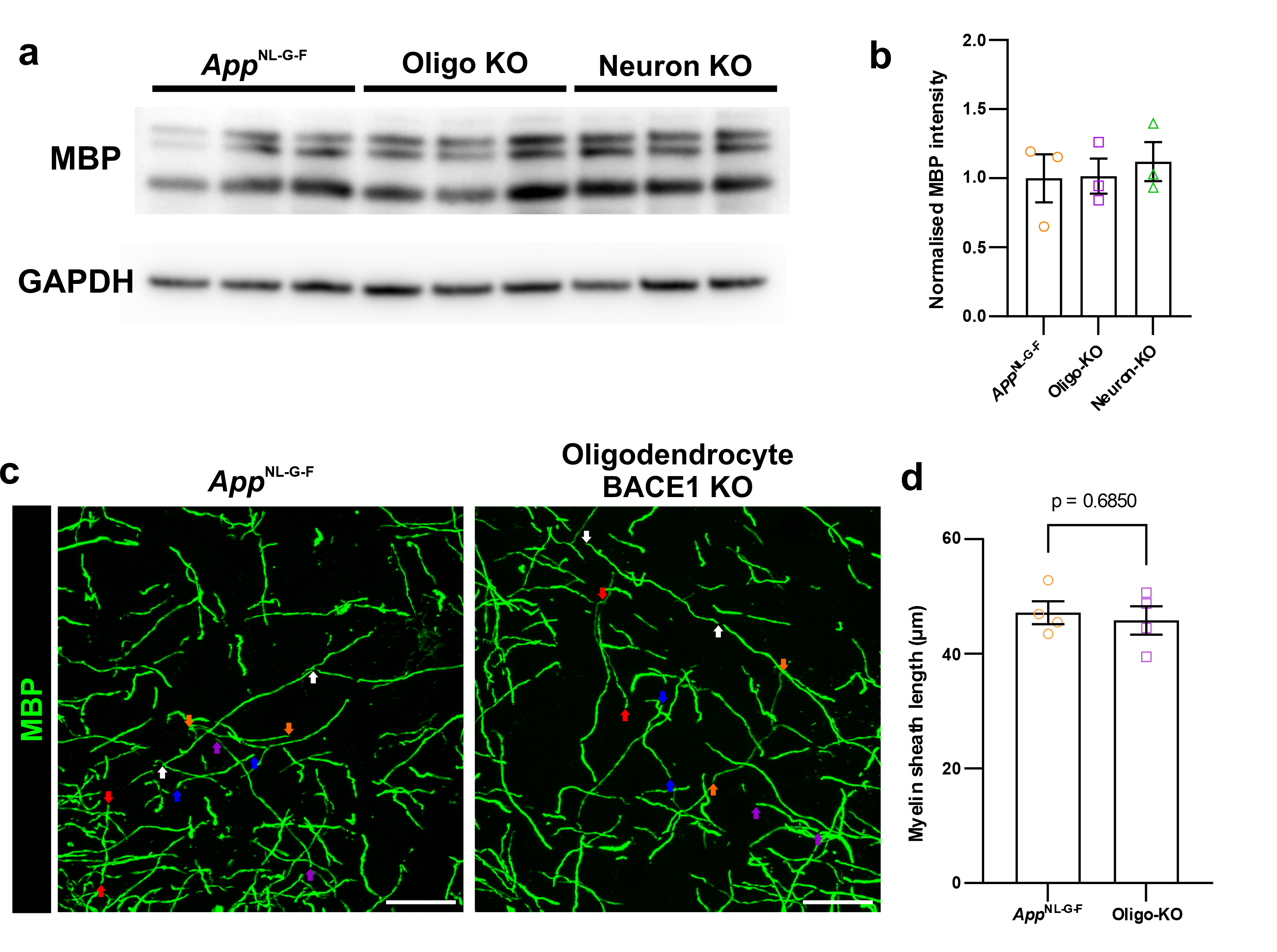

Supplement: S9 Fig — (a) Western blot for MBP (top) and GAPDH loading control (bottom) on forebrain homogenates from AppNL-G-F mice (left 3 lanes), and AppNL-G-F mice with BACE1 knocked out (KO) specifically in oligodendrocytes (Oligo-KO; middle 3 lanes) or neurons (Neuron-KO; right 3 lanes). (b) Quantification of the MBP bands normalised to GAPDH loading control shows no significant difference in MBP levels in mice with BACE1 knocked out. In (b), data points represent individual mice (n = 3 per group) with bars showing mean ± SEM. One-way ANOVA: F(2,6) = 0.1924, p = 0.8299. (c) Representative immunofluorescent images of myelin sheaths (labelled with MBP, green) in the sparsely myelinated Layers 2/3 of the primary somatosensory cortex showing no significant differences between AppNL-G-F mice (left) and AppNL-G-F mice with BACE1 KO specifically in oligodendrocytes (Oligo-KO; right). Coloured pairs of arrows indicate the start and end of example myelin sheaths. Scale bar = 30 μm. (d) Quantification of the lengths of myelin sheaths in the cortex, as previously described [71,72], shows no differences in mice with BACE1 knocked out after 4 weeks of age. In (d), each data point represents the average of 120 myelin sheaths measured from an individual mouse (n = 4 per group) with bars showing mean ± SEM. Unpaired t test: t(6) = 0.4259. Source data are available in S1 Data. (TIF) [file pbio.3002727.s009.tif]

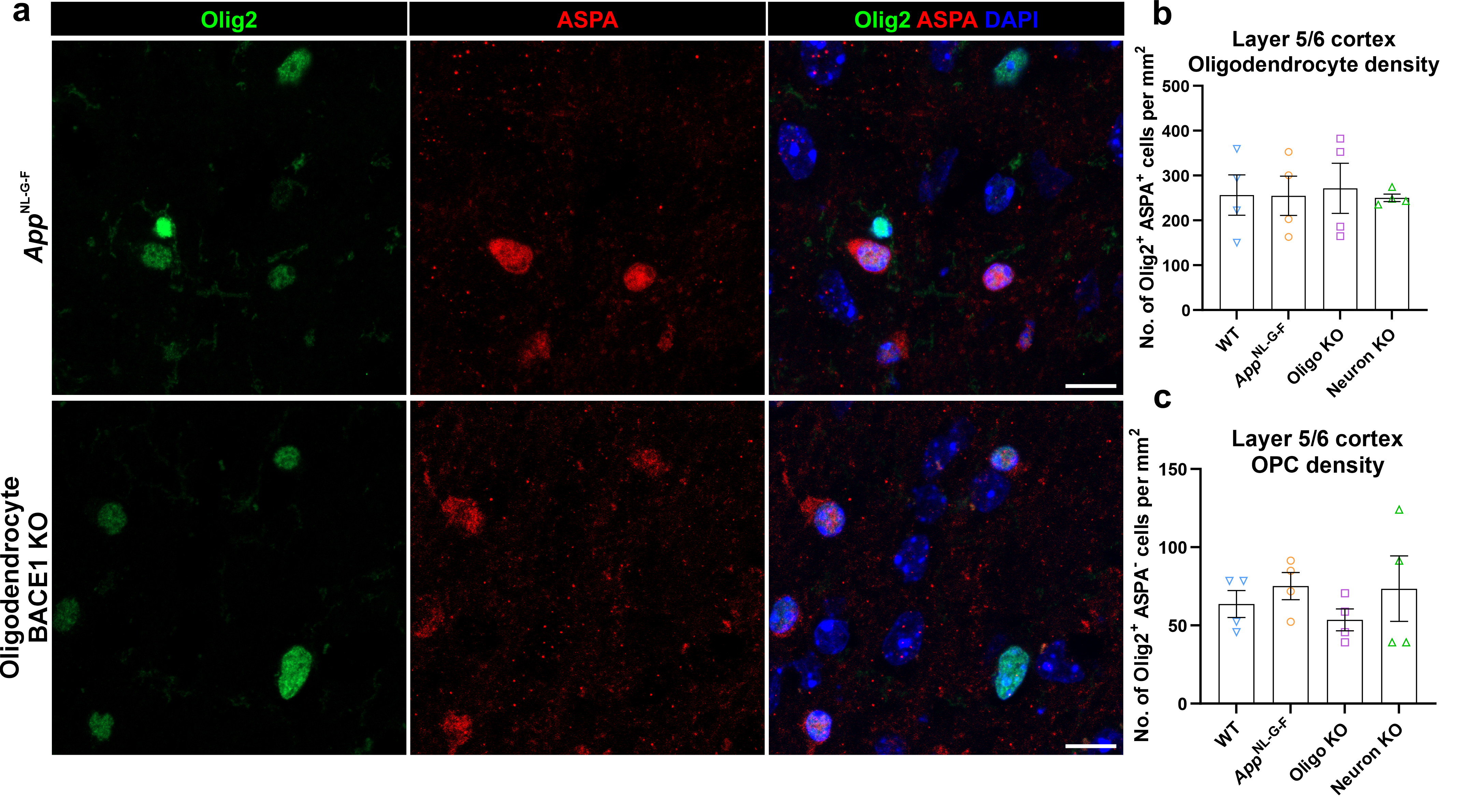

Supplement: S10 Fig — (a) Immunofluorescent images showing pan-oligodendroglial marker Olig2 (green), oligodendrocyte-specific marker ASPA (red), and DAPI (blue) in the cortex of AppNL-G-F control mice (top) and AppNL-G-F mice with BACE1 KO specifically in oligodendrocytes (bottom). Scale bar = 25 μm. (b) Quantification of Olig2+ ASPA+ cells shows no significant difference in oligodendrocyte number in mice with BACE1 KO. (c) Quantification of Olig2+ ASPA- cells shows no significant difference in the number of oligodendrocyte precursor cells (OPCs) in mice with BACE1 KO. In (b and c), data points represent individual mice (n = 4 per group) with bars showing mean ± SEM. One-way ANOVA: F(3,12) = 0.04745, 0.6279; p = 0.9856, 0.6107 in (b and c), respectively. Source data are available in S1 Data. (TIF) [file pbio.3002727.s010.tif]

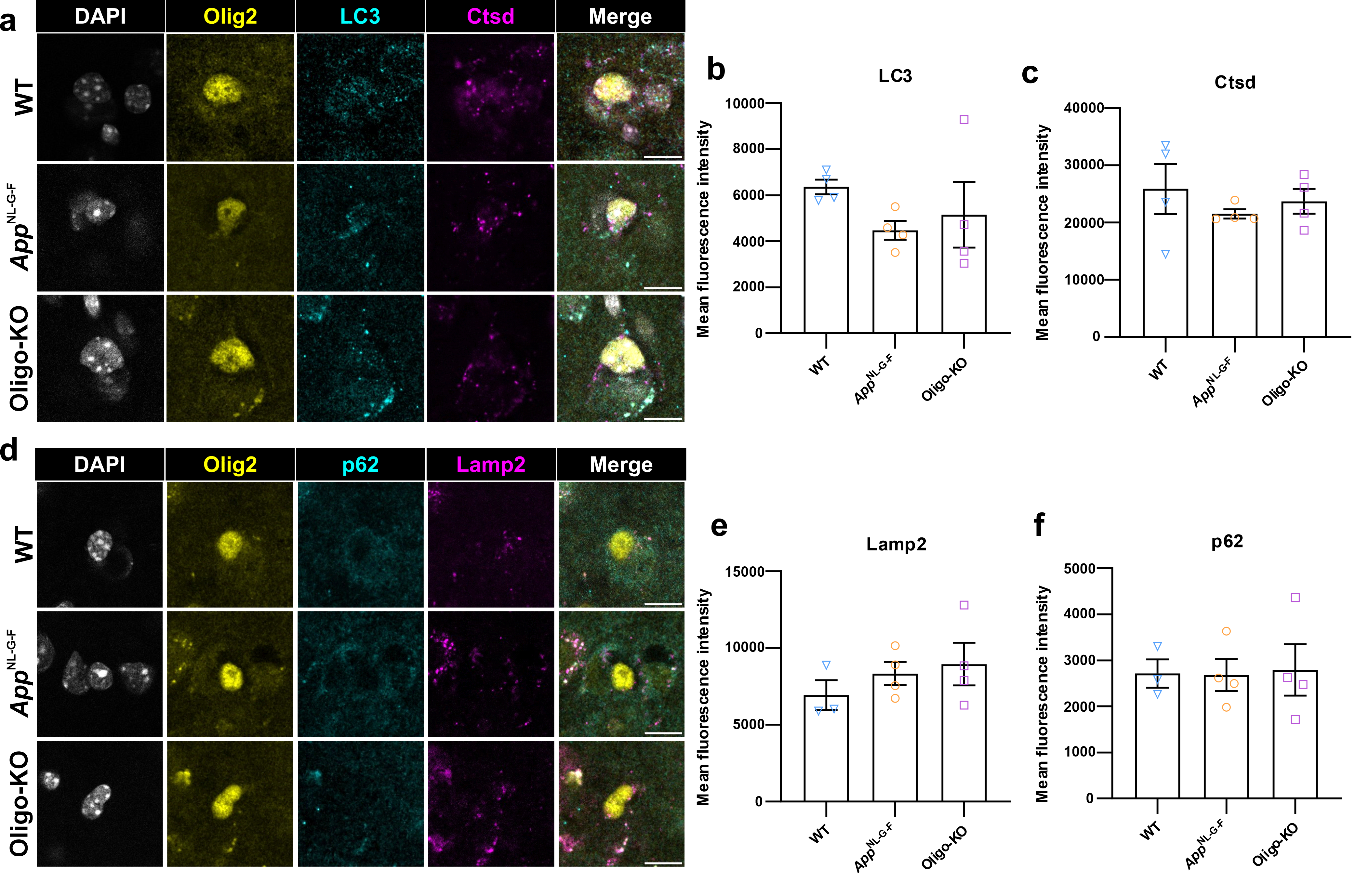

Supplement: S11 Fig — (a) Immunofluorescent images showing oligodendroglial marker Olig2 (yellow), autophagosome marker MAP1LC3B/LC3B (microtubule-associated protein 1 light chain β; LC3; cyan), lysosome protease Cathepsin-d (Ctsd; magenta), and nuclear marker DAPI (white) in the cortex of WT (top), AppNL-G-F control mice (middle), and AppNL-G-F mice with BACE1 KO specifically in oligodendrocytes (Oligo-KO; bottom). (b, c) Quantification shows no significant changes in LC3 (b), or Ctsd (c) within oligodendroglia upon knockout of BACE1 in oligodendrocytes. (d) Immunofluorescent images showing oligodendroglial marker Olig2 (yellow), autophagy adapter Sqstm1/p62 (cyan), lysosome marker Lamp2 (lysosome-associated membrane protein 2; magenta), and nuclear marker DAPI (white) in the cortex of WT (top), AppNL-G-F control mice (middle), and AppNL-G-F mice with BACE1 KO specifically in oligodendrocytes (Oligo-KO; bottom). (e, f) Quantification shows no significant changes in p62 (e), or Lamp2 (f) within oligodendroglia upon knockout of BACE1 in oligodendrocytes. In (b–c and e–f), data points represent individual mice (n = 3 WT in e and f, n = 4 for all other groups) with bars showing mean ± SEM. One-way ANOVA: F(2,9) = 1.207, 0.5826; p = 0.3434, 0.5782 in (b and c), respectively. F(2,8) = 0.7969, 0.01823; p = 0.4835, 0.9820 in (e and f), respectively. Source data are available in S1 Data. (TIF) [file pbio.3002727.s011.tif]

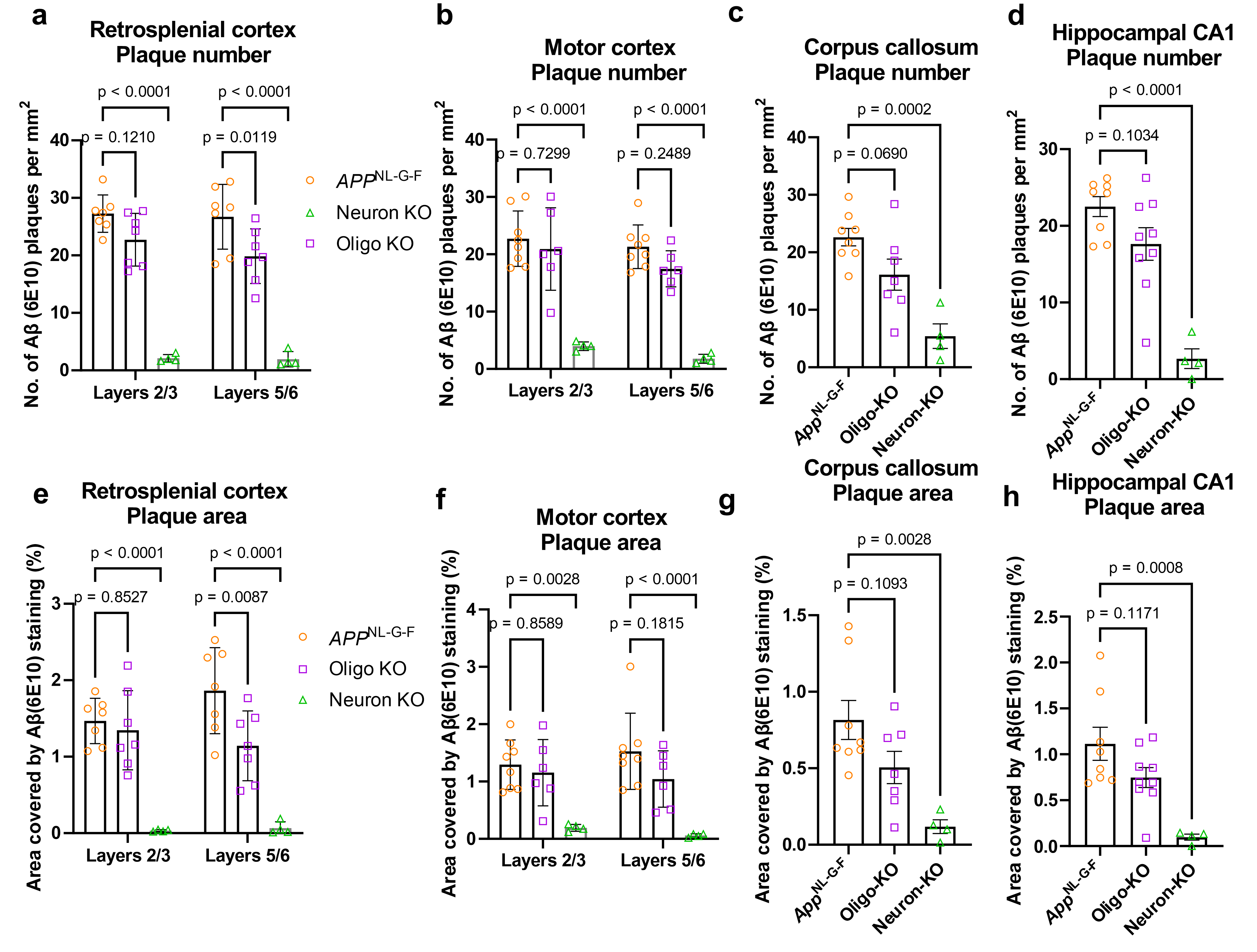

Supplement: S12 Fig — (a, e) Quantification of plaque number (a) and plaque area (e) across different layers of the retrosplenial cortex suggests that oligodendrocyte specific KO of BACE1 leads to a greater reduction in plaque pathology in deeper layers (Layers 5/6) than is observed in superficial layers (Layers 2/3). Data points represent individual mice (n = 7 AppNL-G-F, 7 Oligo-KO, 4 Neuron-KO) with bars showing mean ± SEM. Two-way repeated measures ANOVA with Tukey’s post hoc tests. BACE1 KO effect: F(2,15) = 61.25 (a), 22.59 (e); p < 0.0001 (all); Layer-BACE1 KO interaction effect: F(2,15) = 0.8174 (a), 5.892 (e); p = 0.4603 (a), p = 0.0129 (e). (b, f) Quantification of plaque number (b) and plaque area (f) across different layers of the motor cortex. Data points represent individual mice (n = 8 AppNL-G-F, 6 Oligo-KO, 4 Neuron-KO) with bars showing mean ± SEM. Two-way repeated measures ANOVA with Tukey’s post hoc tests. BACE1 KO effect: F(2,15) = 47.58 (b), 13.24 (f); p < 0.0001 (b), p = 0.0005 (f); Layer-BACE1 KO interaction effect: F(2,15) = 0.2164 (b), 0.9289 (f); p = 0.8079 (b), p = 0.4166 (f). (c, d, g, h) Quantification of plaque number (c, d) and plaque area (g, h) in the corpus callosum (c, g) and hippocampal area CA1 (d, h) shows a trend towards reduction in Oligo-KO mice in these regions. In (c, g), data points represent individual mice (n = 8 AppNL-G-F, 7 Oligo-KO, 4 Neuron-KO) with bars showing mean ± SEM. One-way ANOVA with Dunnet’s post hoc tests: F(2,16) = 12.94, 7.549; p = 0.0005, 0.0049 in (c, g), respectively. In (d, h), data points represent individual mice (n = 8 AppNL-G-F, 9 Oligo-KO, 4 Neuron-KO) with bars showing mean ± SEM. One-way ANOVA with Dunnet’s post hoc tests: F(2,18) = 21.83, 9.338; p < 0.0001, p = 0.0017 in (g, h) respectively. Source data are available in S1 Data. (TIF) [file pbio.3002727.s012.tif]

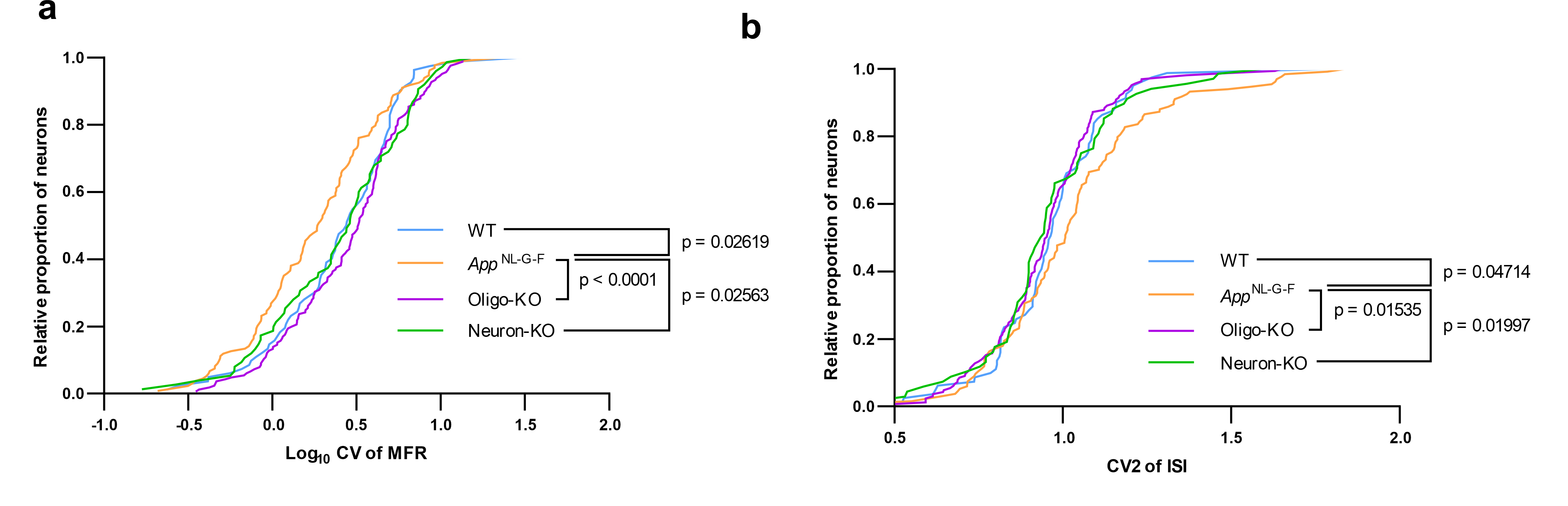

Supplement: S13 Fig — (a) Cumulative frequency distribution of the coefficient of variation (CV) of the mean firing rate (MFR) shows that variability in MFR during rest is restored to WT levels in AppNL-G-F mice with BACE1 knocked out (KO) specifically in oligodendrocytes (Oligo-KO; WT vs. Oligo-KO: p = 0.6192). (b) Cumulative frequency distribution of the coefficient of variation 2 (CV2) of the inter-spike interval (ISI) shows the restoration of spike train variability to WT levels in Oligo-KO mice (WT vs. Oligo-KO: p = 0.5032) during quiescence. In (a), individual neurons/units are plotted (n = 82 WT, 134 AppNL-G-F, 165 Oligo-KO, 75 Neuron-KO) from 4 (WT) or 3 mice per group. Kolmogrov–Smirnov tests (D = 0.2026 (WT vs. AppNL-G-F), 0.2688 (AppNL-G-F vs. Oligo-KO), 0.2088 (AppNL-G-F vs. Neuron-KO)). In (b) individual neurons/units are plotted (n = 81 WT, 134 AppNL-G-F, 165 Oligo-KO, 68 Neuron-KO) from 4 (WT) or 3 mice per group. Kolmogrov–Smirnov tests (D = 0.1891 (WT vs. AppNL-G-F), 0.1787 (AppNL-G-F vs. Oligo-KO), 0.2215 (AppNL-G-F vs. Neuron-KO)). Source data are available in S1 Data. (TIF) [file pbio.3002727.s013.tif]

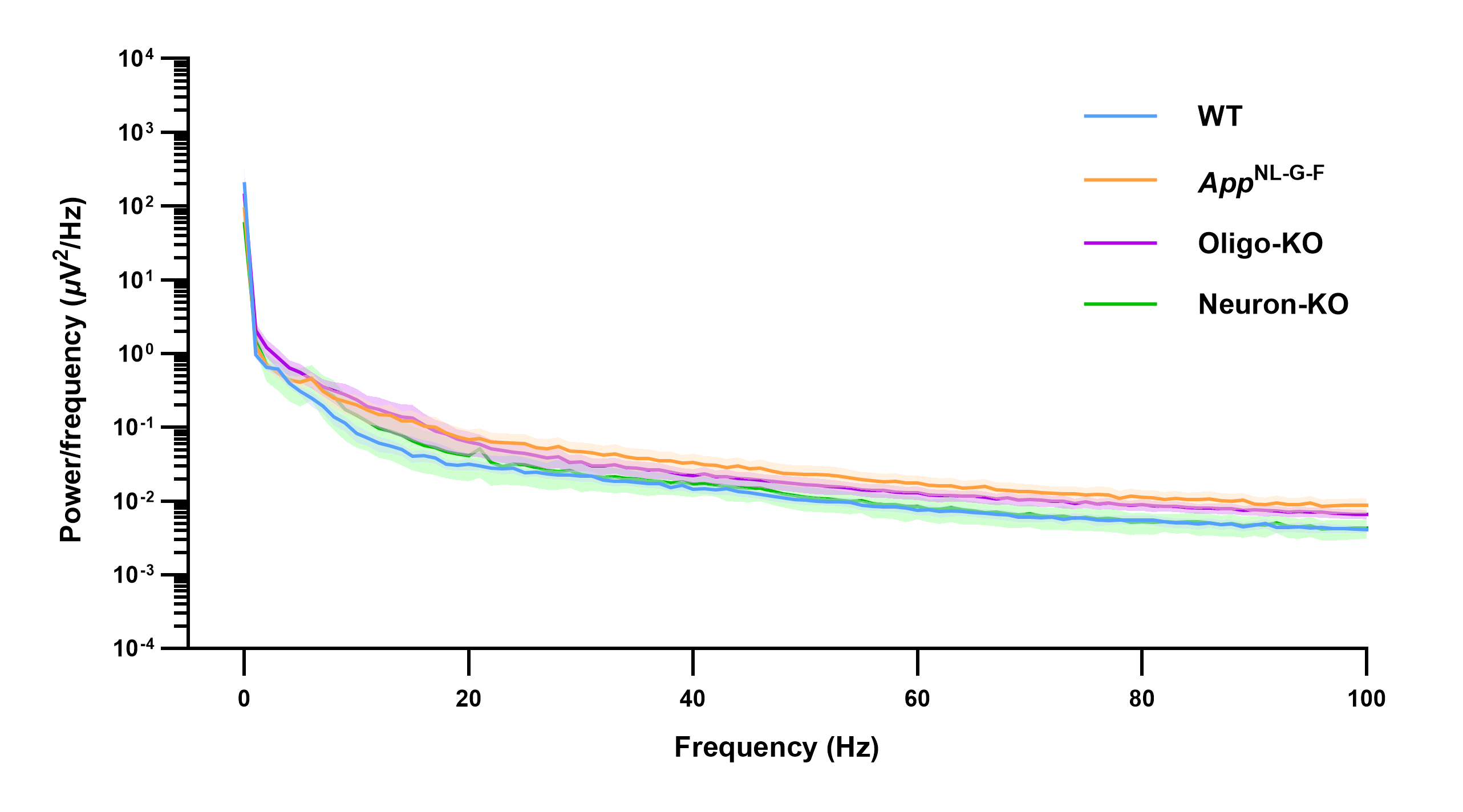

Supplement: S14 Fig — Power spectrum of local field potential (LFP) recordings in retrosplenial cortex showing no major differences between genotypes. n = 4 (WT), 3 (other groups) mice. One-way ANOVA: F(3,9) = 0.6235, p = 0.6175. Source data are available in S1 Data. (TIF) [file pbio.3002727.s014.tif]

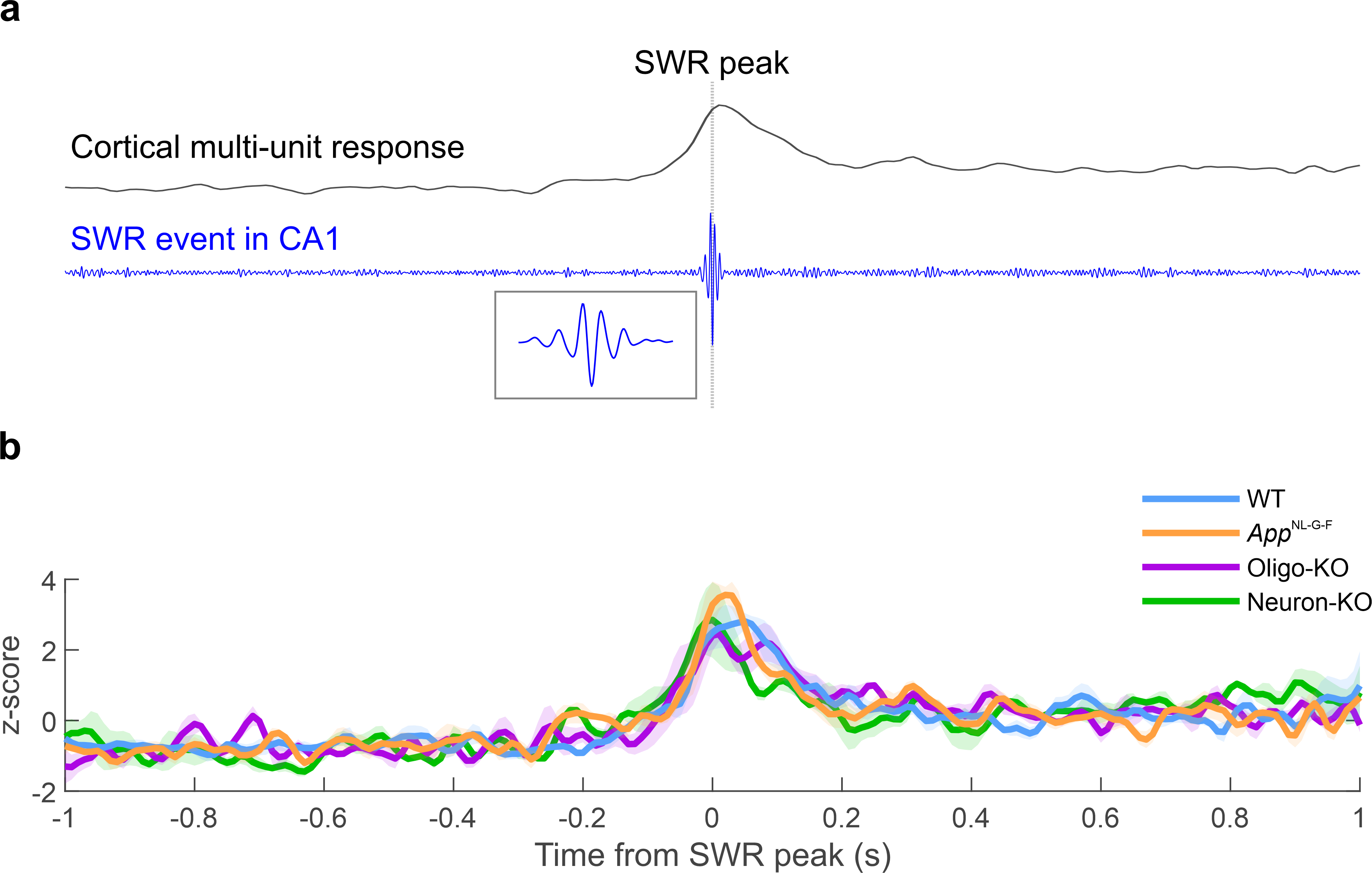

Supplement: S15 Fig — (a) Schematic illustrating a typical cortical multiunit response (top; black) to a sharp wave ripple (SWR) event in CA1 (bottom; blue). (b) Trace of the averaged cortical multiunit responses to CA1 SWR events showing no significant differences between genotypes in amplitude [peak z-score; one-way ANOVA: F(3,8) = 1.008, p = 0.4381] or timing [delay time of centre of mass; one-way ANOVA: F(3,8) = 1.186, p = 0.3745]. n = 4 (WT), 3 (AppNL-G-F, Oligo-KO), 2 (Neuron-KO) mice. Source data are available in S1 Data. (TIF) [file pbio.3002727.s015.tif]

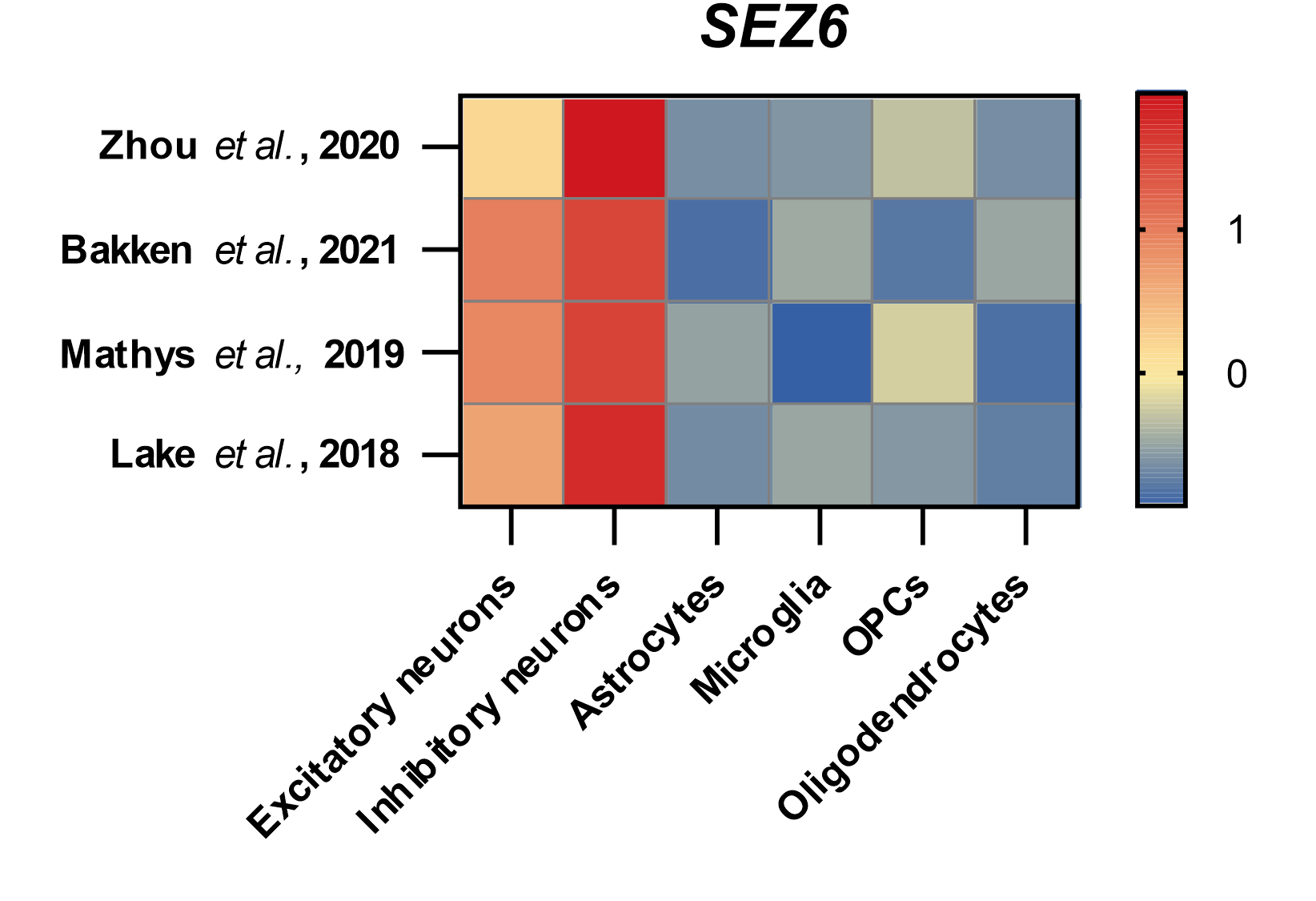

Supplement: S16 Fig — Heatmap showing the log2 (norm count) z-score of SEZ6 across different cell types from 3 different datasets [5,10–12]. [OPCs: oligodendrocyte precursor cells]. (TIF) [file pbio.3002727.s016.tif]
