# Supplementary material for: Selective suppression of oligodendrocyte-derived amyloid beta rescues neuronal dysfunction in Alzheimer’s disease
Source: PLoS Biol. 2024 Jul 23;22(7):e3002727. doi: 10.1371/journal.pbio.3002727 (PMC11265669; doi:10.1371/journal.pbio.3002727)
Supplement: S1 Raw Images — (PDF) [file pbio.3002727.s018.pdf]

S9a Fig, top row
MBP (chemiluminescence)

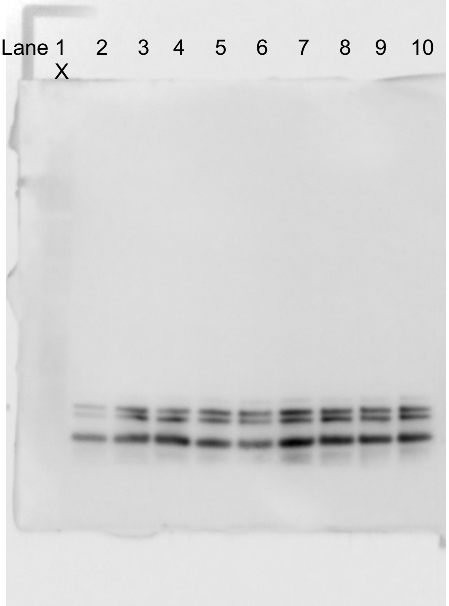

MBP (chemiluminescence with colour imaging of ladder overlaid)

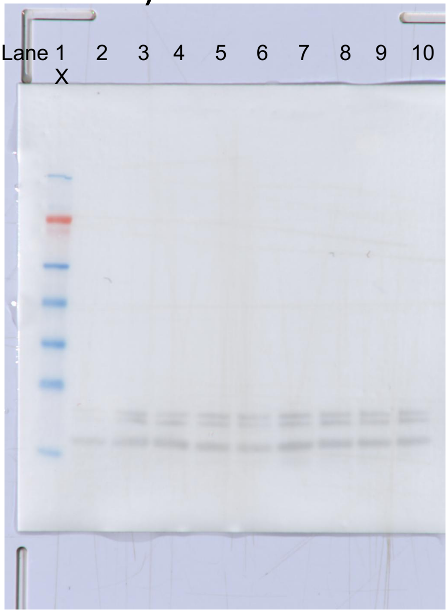

Lane

1 SeeBlue Plus2 Ladder (not shown in figure)
2 *App*$^{NL-G-F}$ mouse 1
3 *App*$^{NL-G-F}$ mouse 2
4 *App*$^{NL-G-F}$ mouse 3
5 Oligo-KO mouse 1
6 Oligo-KO mouse 2
7 Oligo-KO mouse 3
8 Neuron-KO mouse 1
9 Neuron-KO mouse 2
10 Neuron-KO mouse 3

S9b Fig, bottom row
GAPDH (chemiluminescence)

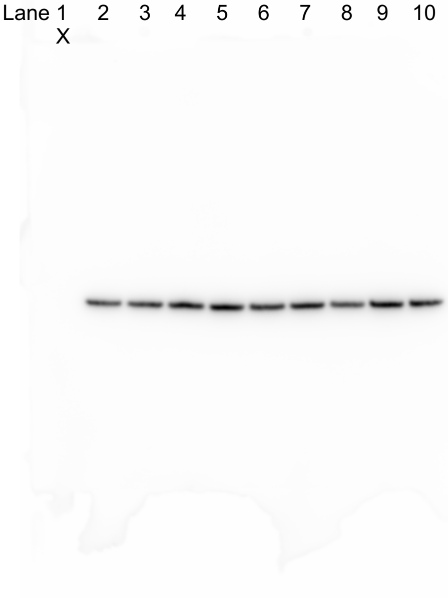

GAPDH (chemiluminescence with colour imaging of ladder overlaid)

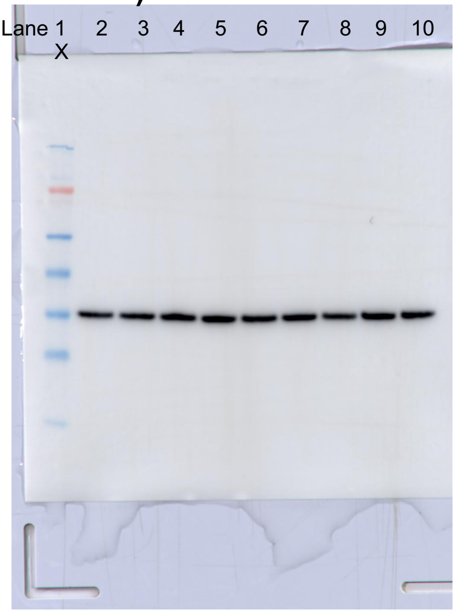

Lane

1 SeeBlue Plus2 Ladder (not shown in figure)
2 *App*$^{NL-G-F}$ mouse 1
3 *App*$^{NL-G-F}$ mouse 2
4 *App*$^{NL-G-F}$ mouse 3
5 Oligo-KO mouse 1
6 Oligo-KO mouse 2
7 Oligo-KO mouse 3
8 Neuron-KO mouse 1
9 Neuron-KO mouse 2
10 Neuron-KO mouse 3

Images captured using Amersham Imager 680
GAPDH is reprobe of same membrane as MBP, after stripping
