# Supplementary material for: Selective suppression of oligodendrocyte-derived amyloid beta rescues neuronal dysfunction in Alzheimer’s disease
Source: PLoS Biol. 2024 Jul 23;22(7):e3002727. doi: 10.1371/journal.pbio.3002727 (PMC11265669; doi:10.1371/journal.pbio.3002727)
Supplement: Peer review history

# Editor report: Decision Letter 0

**DOI:** 10.1371/journal.pbio.3002727.r001

**Authors:** Christian Schnell, PhD

28 Jun 2024

Dear Marc, 

Thank you for submitting your manuscript entitled "Selective suppression of oligodendrocyte-derived amyloid beta rescues neuronal dysfunction in Alzheimer's Disease" for consideration as a Research Article by PLOS Biology.

Your manuscript has now been evaluated by the PLOS Biology editorial staff as well as by an academic editor with relevant expertise and I am writing to let you know that we would like to move forward with your manuscript with the aim of publishing it in PLOS Biology. 

As you have disclosed during the submission, your manuscript was already reviewed elsewhere and you opted into portable peer review. Based on our Academic Editor's assessment of the rebuttal letter and revisions of your manuscript, we are forgoing further rounds of peer review.

I would encourage you to opt into publishing the peer-review process file. While we will not be able to publish the peer reviewer reports from the previous rounds of review, this ensures that the editorial decision letters will be published.

While we can do some of the editorial checks only after the full submission of your manuscript, below are already some of the editorial points that we will ask you to address:

* Please group all of the reviewers reports and your responses into one file and upload it as a "prior peer reviews" file. 

* Please include the full name of the IACUC/ethics committee that reviewed and approved the animal care and use protocol/permit/project license. Please also include an approval number.

* Please include information about the form of consent (written/oral) given for research involving human participants. All research involving human participants must have been approved by the authors' Institutional Review Board (IRB) or an equivalent committee, and must have been conducted according to the principles expressed in the Declaration of Helsinki.

* DATA POLICY:

You may be aware of the PLOS Data Policy, which requires that all data be made available without restriction: http://journals.plos.org/plosbiology/s/data-availability. For more information, please also see this editorial: http://dx.doi.org/10.1371/journal.pbio.1001797

Note that we do not require all raw data. Rather, we ask that all individual quantitative observations that underlie the data summarized in the figures and results of your paper be made available in one of the following forms:

1) Supplementary files (e.g., excel). Please ensure that all data files are uploaded as 'Supporting Information' and are invariably referred to (in the manuscript, figure legends, and the Description field when uploading your files) using the following format verbatim: S1 Data, S2 Data, etc. Multiple panels of a single or even several figures can be included as multiple sheets in one excel file that is saved using exactly the following convention: S1_Data.xlsx (using an underscore).

2) Deposition in a publicly available repository. Please also provide the accession code or a reviewer link so that we may view your data before publication. 

Regardless of the method selected, please ensure that you provide the individual numerical values that underlie the summary data displayed in the following figure panels as they are essential for readers to assess your analysis and to reproduce it: 2BCD, 3BCDFG, 4BC, 5BD, S2, S3ABCDEGHI, S4ABCD, S5ADEF and further similar panels in the supplementary information. 

NOTE: the numerical data provided should include all replicates AND the way in which the plotted mean and errors were derived (it should not present only the mean/average values).

Please also ensure that figure legends in your manuscript include information on where the underlying data can be found, and ensure your supplemental data file/s has a legend.

Please ensure that your Data Statement in the submission system accurately describes where your data can be found.

* CODE POLICY

Per journal policy, if you have generated any custom code during the course of this investigation, please make it available without restrictions. Please ensure that the code is sufficiently well documented and reusable, and that your Data Statement in the Editorial Manager submission system accurately describes where your code can be found. 

Please note that we cannot accept sole deposition of code in GitHub, as this could be changed after publication. However, you can archive this version of your publicly available GitHub code to Zenodo. Once you do this, it will generate a DOI number, which you will need to provide in the Data Accessibility Statement (you are welcome to also provide the GitHub access information). See the process for doing this here: https://docs.github.com/en/repositories/archiving-a-github-repository/referencing-and-citing-content

* BLOT AND GEL REPORTING REQUIREMENTS:

We require the original, uncropped and minimally adjusted images supporting all blot and gel results reported in an article's figures or Supporting Information files. We will require these files before a manuscript can be accepted so please prepare and upload them now. Please carefully read our guidelines for how to prepare and upload this data: https://journals.plos.org/plosbiology/s/figures#loc-blot-and-gel-reporting-requirements

Please login to Editorial Manager where you will find the paper in the 'Submissions Needing Revisions' folder on your homepage. Please click 'Revise Submission' from the Action Links and complete all additional questions in the submission questionnaire.

Once your full submission is complete, your paper will undergo a series of checks . After your manuscript has passed the checks it will be checked by the editor. To provide the metadata for your submission, please Login to Editorial Manager (https://www.editorialmanager.com/pbiology) within two working days, i.e. by Dec 25 2024 11:59PM.

Feel free to email us at plosbiology@plos.org if you have any queries relating to your submission.

Kind regards,

Christian

Christian Schnell, PhD

Senior Editor

PLOS Biology

cschnell@plos.org

# Editor report: Decision Letter 1

**DOI:** 10.1371/journal.pbio.3002727.r002

**Authors:** Christian Schnell, PhD

1 Jul 2024

Dear Marc,

Thank you for the submission of your revised Research Article "Selective suppression of oligodendrocyte-derived amyloid beta rescues neuronal dysfunction in Alzheimer's Disease" for publication in PLOS Biology. On behalf of my colleagues and the Academic Editor, Mikael Simons, I am pleased to say that we can in principle accept your manuscript for publication, provided you address any remaining formatting and reporting issues. These will be detailed in an email you should receive within 2-3 business days from our colleagues in the journal operations team; no action is required from you until then. Please note that we will not be able to formally accept your manuscript and schedule it for publication until you have completed any requested changes.

When addressing the requests coming from the journal operations team, please also address the following issues:

* Please add links to the funding agencies' websites in the Financial Disclosure statement in the manuscript details

* Please modify the abstract to specify that the study contains data from human brains and mouse models. For example: "Reduction of amyloid beta (Aβ) has been shown to be effective in treating Alzheimer’s Disease (AD), but the underlying assumption that neurons are the main source of pathogenic Aβ is untested. Here we challenge this prevailing belief by demonstrating that oligodendrocytes are an important source of Aβ in the human brain, and play a key role in promoting abnormal neuronal hyperactivity in AD. We show that selectively suppressing oligodendrocyte Aβ production improves AD brain pathology and restores neuronal function in vivo in a mouse model of AD. Our findings suggest that targeting oligodendrocyte Aβ production could be a promising therapeutic strategy for treating AD."

* In the data availability statement in Editorial Manager, please specify which restrictions to data access apply and how they can be assessed. Furthermore, the link in the data availability statement (https://www.ucl.ac.uk/library/openscience-research-support/research-data-management/ucl-research-data-repository) is not valid. Once you have the correct link, please provide it here and specify how the data can be accessed and if there are any restrictions. Please also provide the link to the zenodo repository here. 

* Please modify the COI statement and say that "The other authors have declared that no competing interests exist." (If that's correct).

Please take a minute to log into Editorial Manager at http://www.editorialmanager.com/pbiology/, click the "Update My Information" link at the top of the page, and update your user information to ensure an efficient production process.

PRESS

We frequently collaborate with press offices. If your institution or institutions have a press office, please notify them about your upcoming paper at this point, to enable them to help maximise its impact. If the press office is planning to promote your findings, we would be grateful if they could coordinate with biologypress@plos.org. If you have previously opted in to the early version process, we ask that you notify us immediately of any press plans so that we may opt out on your behalf.

We also ask that you take this opportunity to read our Embargo Policy regarding the discussion, promotion and media coverage of work that is yet to be published by PLOS. As your manuscript is not yet published, it is bound by the conditions of our Embargo Policy. Please be aware that this policy is in place both to ensure that any press coverage of your article is fully substantiated and to provide a direct link between such coverage and the published work. For full details of our Embargo Policy, please visit http://www.plos.org/about/media-inquiries/embargo-policy/.

Thank you again for choosing PLOS Biology for publication and supporting Open Access publishing. We look forward to publishing your study. 

Sincerely, 

Christian

Christian Schnell, PhD

Senior Editor

PLOS Biology

cschnell@plos.org
